# Dental Deformities

**Published:** 1856-01

**Authors:** 


### Dental Abstracts.

The October No. of *American Journal of Dental Science* gives an article from Dr. Levison, of England, on Dental Deformities, and relates two or three cases of constitutional disturbance, as the result of a too crowded condition of the teeth. The practice he adopts, of separating the teeth to relieve the pain, is perhaps not in accordance with the views of many American dentists, yet, when extraction of one or two teeth would not be consented to, it would certainly be advantageous. We give the cases reported:

"Some years since, during my residence at Doncaster, in Yorkshire, a young lady consulted me, having suffered for three or four years constant tooth-ache, which was more or less intense and aggravated by moral causes, on any slight derangement of the stomach. She said, 'I have had the advice of physicians, surgeons and dentists, and without any benefit, nay, I begin to despair of any mitigation.' She was about twenty-five or six years old, a nervo-sanguineous temperament, with a well formed chest and brain, and the appearance of a good natural constitution; and her intellectual and moral attributes had been cultivated, so that, besides being highly intelligent, she was urbane, amiable, and practically benevolent.

"When she called, she appeared much excited, her face was flushed, and her eyes more brilliant than they would be in her ordinary state; evidently arising, however, from cerebral irritation, but whether from any local cause, or from reflex nervous action, or from the vigilance and restlessness induced by her chronic tooth-ache, were in the first instance matter of speculation. As is my wont in obscure cases, I subjected my patient to a rigid cross-examination, inspected afterwards her mouth, and then formed my *diagnosis*, and told the patient I had no doubt that I could soon give her relief. To which she replied, probably more candidly than courteously, 'this I do not anticipate, although I came to you under a forlorn hope that you could mitigate my sufferings.' Before proceeding with the simple treatment and immediate success, it is well to state it was evident that great care had been bestowed during the second dentition; the anterior arches were well formed, and the teeth beautifully arranged in their natural order, but so closely packed together, that it was impossible to pass between them the thinnest thread or silk; and at the posterior ends of the lower jaw the *dentes sapientiœ* were forced under the interior ridge of the coronoid process; and on the upper the same teeth were forced out of the curve, being pressed outwards toward the cheeks.

"I proposed extracting a tooth from each jaw, but, though a sensible woman, she objected, from a notion that it would be removing the key-stone of the arch, and that some marked alteration would take place to mar the symmetry of the teeth, and as a consequence the expression of her mouth.

"Now, as she was a very handsome personage, this line of argument seemed most natural, and formed a sufficient reason for a negative! Yet, as my own conviction was, *a priori*, that all her suffering arose from dental pressure, I next suggested that she should permit me to separate her teeth by means of a dividing file—to this she put in a demurrer, to which I replied, proving that very little of the enamel would be removed (as the file was not thicker than writing-paper), and the operation might be done with perfect impunity. This argument, after due consideration, was admitted to be valid, and the lady assented.

"I then divided every two teeth, making just enough space so as to admit a piece of thread to pass without resistance or obstruction.

"Never shall I forget either her expression or her gratitude. As soon as my operation was over, she said, 'Oh, sir, I have lost the painful symptoms! I have not felt so easy for years.' So great was her joy, that tears filled her eyes. I saw her repeatedly afterwards, and she had not any return of tooth-ache. Her features, which were of a classic form, assumed their natural sweet expression, her health greatly improved, and she could continue, without any sensation of lassitude, her passion for literature, and this simply because her sleep was sound and the cerebral functions normal.

"I am tempted to give one more from my note-book, as offering some matter for reflection and comment.

"Mr. P——, a fine, tall, athletic young man, had been for two or three years a soldier and bought off by his relatives, subsequently became a teacher of gymnastics in Brighton. He was above six feet two inches in height, with an enormous chest and upper extremities, and whose muscular system presented one of the most perfectly developed models of strength, from their dense and iron-hardness; yet he suffered from tooth-ache, and came with a dolorous face to ask me to stop him some teeth.

"On looking into his capacious mouth,* I perceived two and thirty teeth, without a speck or blemish—the jaws were ample, the teeth arranged in curves, and the palatal bones finely arched. I told him he had not a tooth that required stopping. He said that this must be an error, as he had suffered tooth-ache more or less for more than two years. It appeared that this, like the preceding case, arose from the close proximity of the teeth, which were jammed together, hence the chronic tooth-ache he had endured. In Mr. P—'s case I did not propose extracting any teeth, but I separated a half dozen in each jaw, and had the satisfaction to have my patient declare that he was free from pain, as if by magic! He sat for a few minutes champing his enormous jaws together, and with a merry voice he made the following statement: 'I seem as if released from some strong controling influence; for I can now move my jaws easily and without pain. But for a very long time my sensations have been as if I wanted to grind the teeth together, to git rid of the irritation, and my gums were always hot and itching, which made me desire to rub something hard against them. But now I'm all right!' Probably the most scientific exposition could not render more graphic the inconvenience and annoyance which he endured from the undue pressure on the dental arches. This condition had interfered with his comfort and affected his spirits. And it may be added, that the chronic inflammatory action, though it did not induce caries in his large and filbert-shaped teeth, yet disease would soon have been developed, judging from their color, but which tendency succumbed to the operation, as the teeth soon became white and brilliantly polished, as there was removed that under-pressure which had interfered with their healthy and normal functions; and he often showed me, with some exultation, that they were not afterwards affected by either local or remote disturbing causes."

A late number of the *News Letter* gives a new method of using gold foil, by Prof. Arthur, which he considers of great

*I took models of Mr. P——'s upper and under jaws, and will send them, with the casts figured in my last communication, for the museum of the Baltimore College, when there is any chance of their going safely.
J. L. L.

